# Phylogenomic Test of the Hypotheses for the Evolutionary Origin of Eukaryotes

**DOI:** 10.1093/molbev/mst272

**Published:** 2014-01-07

**Authors:** Nicolas C. Rochette, Céline Brochier-Armanet, Manolo Gouy

**Affiliations:** ^1^Laboratoire de Biométrie et Biologie Évolutive, CNRS UMR5558, Université de Lyon, Universite Claude Bernard Lyon 1, Villeurbanne, France

**Keywords:** eukaryogenesis, archaea, evolution, phylogeny, tree of life, horizontal gene transfer

## Abstract

The evolutionary origin of eukaryotes is a question of great interest for which many different hypotheses have been proposed. These hypotheses predict distinct patterns of evolutionary relationships for individual genes of the ancestral eukaryotic genome. The availability of numerous completely sequenced genomes covering the three domains of life makes it possible to contrast these predictions with empirical data. We performed a systematic analysis of the phylogenetic relationships of ancestral eukaryotic genes with archaeal and bacterial genes. In contrast with previous studies, we emphasize the critical importance of methods accounting for statistical support, horizontal gene transfer, and gene loss, and we disentangle the processes underlying the phylogenomic pattern we observe. We first recover a clear signal indicating that a fraction of the bacteria-like eukaryotic genes are of alphaproteobacterial origin. Then, we show that the majority of bacteria-related eukaryotic genes actually do not point to a relationship with a specific bacterial taxonomic group. We also provide evidence that eukaryotes branch close to the last archaeal common ancestor. Our results demonstrate that there is no phylogenetic support for hypotheses involving a fusion with a bacterium other than the ancestor of mitochondria. Overall, they leave only two possible interpretations, respectively, based on the early-mitochondria hypotheses, which suppose an early endosymbiosis of an alphaproteobacterium in an archaeal host and on the slow-drip autogenous hypothesis, in which early eukaryotic ancestors were particularly prone to horizontal gene transfers.

## Introduction

All known cellular organisms belong to one of three domains: Bacteria, Archaea, or Eukarya. These three groups not only share common ancestry but also harbor distinctive features. Bacteria and Archaea differ in their replication machineries (Grabowski and Kelman 2003), gene regulation systems (Reeve 2003), membrane chemistry (Pereto et al. 2004; Guldan et al. 2011; Shimada and Yamagishi 2011), and cell wall structure (Kandler and König 1998; Albers and Meyer 2011), among other things. Intriguingly, Eukarya are similar to Archaea for some systems (e.g., the replication, transcription, and translation apparatuses [Reeve 2003; Allers and Mevarech 2005]) and to Bacteria for others (e.g., metabolism [Rivera et al. 1998; Canback et al. 2002] and membrane chemistry [Pereto et al. 2004]). They also possess numerous specific systems that confer them an incomparable cellular complexity: the last eukaryotic common ancestor (LECA) is thought to have had a modern nucleus (Mans et al. 2004) and associated features, such as nuclear pore complexes (Bapteste et al. 2005; Neumann et al. 2010), chromatin (Iyer et al. 2008), linear chromosomes and centromeres (Cavalier-Smith 2010b), nucleolus (Staub et al. 2004), capped and polyadenylated mRNA, and introns (Collins and Penny 2005). It also had mitochondria (which are derived alphaproteobacteria; Embley and Martin 2006; Gabaldón and Huynen 2007), a cytoskeleton based on microtubules and actin (Yutin et al. 2009; Hammesfahr and Kollmar 2012), a complete vesicle and membrane-trafficking system allowing for endocytosis (Dacks et al. 2009; Yutin et al. 2009; De Craene et al. 2012), a modern cell cycle (Eme et al. 2011), and a sexual cycle (meiosis [Ramesh et al. 2005] and syngamy).

Because of their elaborate cellular biology and their peculiar mosaicism and also because we are ourselves eukaryotes, the origin of Eukarya has drawn much attention. Many diverse hypotheses have been proposed, reflecting the profound disagreements among their authors over what evolutionary events should or should not be considered possible (see Embley and Martin [2006] for a review). These hypotheses can be classified into three main classes. In “autogenous” hypotheses, the eukaryotic endomembrane system and nucleus evolved spontaneously, subsequently making possible the mitochondrial endosymbiosis (Doolittle 1978; Cavalier-Smith 2002; Jékely 2003; Lester et al. 2006; de Duve 2007; Cavalier-Smith 2010b; Devos and Reynaud 2010; Küper et al. 2010; Forterre 2011; Poole and Neumann 2011; Martijn and Ettema 2013). Conversely, “early-mitochondria” hypotheses propose that the evolution of cellular complexity was triggered by a primordial endosymbiosis of an alphaproteobacterium into an archaeal host (Martin and Müller 1998; Vellai et al. 1998; Searcy 2003). Finally, “ternary” hypotheses advocate that the organism that engulfed the ancestor of mitochondria was itself a chimera of two prokaryotes (Margulis et al. 2000; Godde 2012). Among popular ternary hypotheses are the “endokaryotic” hypotheses in which the nucleus derives from an archaeon while the cytoplasm derives from a bacterium (Lake and Rivera 1994; Gupta and Golding 1996; Horiike et al. 2004; Lopez-Garcia and Moreira 2006).

All these hypotheses for the origin of Eukarya imply assumptions regarding the lineages that were involved in this process. In each case, these lineages are believed to have contributed to the modern eukaryotic genome, be it by vertical descent, endosymbiotic gene transfer (EGT; a process well known for the mitochondrion [Embley and Martin 2006]) or other forms of horizontal gene transfer (HGT). These hypotheses are therefore associated with different phylogenomic predictions, which can be tested by means of molecular phylogeny. We hereafter give a few representative examples. The “syntrophy hypothesis” (Lopez-Garcia and Moreira 2006), an endokaryotic hypothesis, proposes that Eukarya are a chimera between a methanogen (thus a euryarchaeon [Gribaldo and Brochier-Armanet 2006]) and a deltaproteobacterium, hosting an alphaproteobacterial endosymbiont. Therefore, it predicts that ancestral eukaryotic genes, when they have prokaryotic homologs, should be related to euryarchaeal, deltaproteobacterial, and alphaproteobacterial genes. Similarly, according to the “hydrogen hypothesis” (Martin and Müller 1998), an early-mitochondria hypothesis, ancestral eukaryotic genes are expected to derive from the alphaproteobacterial ancestor of mitochondria and from the methanogenic euryarchaeon that hosted it. Finally, among autogenous hypotheses proponents, the Neomura hypothesis (Cavalier-Smith 2010b) assumes that Eukarya are the sister group of all Archaea and explains the existence of (apparently) bacteria-related genes in Eukarya by EGTs from the mitochondrion and by massive losses by the ancestors of Archaea of genes that existed in the last universal common ancestor (LUCA), so that Eukarya and Bacteria share genes Archaea lack. Other autogenous hypotheses propose that Eukarya stem from within Archaea but have undergone a massive acquisition of bacterial genes, either by EGT or HGT from diverse lineages (Lester et al. 2006; Martijn and Ettema 2013). The slow-drip hypothesis, for instance, advocates that early eukaryotic ancestors acquired many new genes through HGT, like prokaryotes do today (Lester et al. 2006).

Given these contrasting predictions, investigating the phylogenetic relationships between eukaryotic and prokaryotic genes on a genomic scale is an essential piece in the puzzle of the origin of eukaryotes. This question was addressed several times with diverse approaches, including ones based on Blast or similar tools (Horiike et al. 2001; Esser et al. 2004; Atteia et al. 2009; Koonin 2010; Szklarczyk and Huynen 2010), circular genome-content graphs (Rivera and Lake 2004), dekapentagonal maps (Zhaxybayeva et al. 2004), iterated supertrees (Pisani et al. 2007), as well as strategies based on the parallel analysis of many single-gene phylogenies (Saruhashi et al. 2008; Yutin et al. 2008; Thiergart et al. 2012), which also differ greatly in the way the data were collected and processed. All studies agree that the eukaryotic genome is a mosaic of archaea-related, bacteria-related, and eukaryotic-specific genes, with bacteria-related genes somewhat outnumbering archaea-related genes. At taxonomic levels finer than domains, in contrast, the picture is confused. Recent studies (Pisani et al. 2007; Saruhashi et al. 2008; Thiergart et al. 2012) have detected a connection to Alphaproteobacteria, but along with strong signals to other bacterial groups (not necessarily the same ones in different studies). Several interpretations can explain this pattern, which have not been disentangled. Results regarding archaea-related eukaryotic genes have also been ambiguous (Gribaldo et al. 2010). Some studies argued for a sister relationship between Eukarya and Archaea (Brown et al. 2001; Ciccarelli et al. 2006; Yutin et al. 2008), others for a branching of Eukarya deep within Archaea (Rivera and Lake 2004; Saruhashi et al. 2008; Guy and Ettema 2011; Williams et al. 2012) and yet others for a shallow, within-Euryarchaeota branching (Pisani et al. 2007; Thiergart et al. 2012).

We dissected the origins of eukaryotic genes in much more detail than previous studies. In particular, we distinguished between genes whose phylogeny actually supports a relationship between eukaryotes and a particular prokaryotic taxonomic group, genes whose evolutionary histories are blurred by HGTs among prokaryotes, and genes that hold little phylogenetic signal. We show that the set of genes that link to alphaproteobacteria essentially consists of genes involved in mitochondrial respiration and protein processing. Furthermore, there exists no support for the involvement of a particular bacterial lineage other than Alphaproteobacteria in the origin of Eukarya. Most bacteria-related eukaryotic genes cannot be traced to a specific taxonomic group, in many cases because of HGT among Bacteria but sometimes because of lack of signal. Lastly, the analysis of archaea-related genes support that Eukarya branch near the root of Archaea, either deep within them or as a close outgroup. These findings contradict many of the existing hypotheses regarding the origin of eukaryotes.

## Results

### Identification of LECA Clades, Phylogenetic Inferences, and Taxonomic Sampling

The HOGENOM (v5) database contains clusters of homologous sequences built from 946 complete genomes from the three domains of life (Penel et al. 2009). From this database, we retrieved 665 clusters of homologs that contained sequences of diverse Eukarya, plus Archaea or/and Bacteria. On the basis of maximum likelihood (ML) trees of these clusters, we identified all monophyletic groups of eukaryotic sequences that could be traced back to LECA (hereinafter “LECA clades”). In 409 of the 665 clusters of homologs, exactly one LECA clade was identified. In 65 clusters of homologs, two to four distinct LECA clades were identified. These cases typically correspond to genes existing in both cytoplasmic and mitochondrial version, such as some of the ribosomal proteins. In the remaining 191 clusters of homologs, no LECA clade existed because eukaryotic sequences were polyphyletic. Altogether we identified 554 LECA clades. Each LECA clade corresponds to one gene in the genome of LECA, except when gene duplications occurred on the stem branch of eukaryotes, in which case one LECA clade may correspond to several paralogs in the genome of LECA.

The next step was to determine the relationships between each LECA clade and its archaeal and/or bacterial homologs through accurate phylogenetic reconstructions. Because the initial trees were large (670 sequences on average) and taxonomically unbalanced (reflecting the taxonomic biases in genome sequencing projects), we selected 144 and 39 representative genomes for Bacteria and Archaea, respectively (table 1), and ten representative sequences for each LECA clade. This reduced the average number of sequence per tree to 115. We made independent ML phylogenetic reconstructions for each of the 554 LECA clades. 434 LECA clades had more than 50% nonparametric bootstrap support for monophyly and were retained, while those with a lower support were considered to be ambiguous and not analyzed further.

**Table 1.** Taxonomic Distribution of Selected Archaeal and Bacterial Species, and Minimal Number of Representatives Required by the Corresponding Configurations.

Group	Sampling	Threshold
Acidobacteria	3	3
Actinobacteria	15	Half^a^
Alphaproteobacteria	10	Half
Aquificae	4	3
Bacilli	9	Half
Bacteroidetes	15	Half
Betaproteobacteria	4	3
Chlamydiae	3	3
Chlorobi	5	4
Chloroflexi	5	4
Clostridia	9	Half
Crenarchaeota	11	Half
Cyanobacteria	15	Half
Deinococcus-thermus	2	.^b^
Deltaproteobacteria	8	Half
Dictyoglomi	1	.
Elusimicrobia	2	.
Epsilonproteobacteria	5	3
Euryarchaeota	25	Half
Fusobacteria	1	.
Gammaproteobacteria	7	Half
Gemmatimonadetes	1	.
Korarchaeota	1	.
Mollicutes	4	3
Nitrospirae	1	.
Planctomycetes	3	3
Spirochaetes	4	3
Thaumarchaeota	2	.
Thermotogae	4	3
Uncl. proteobacteria	1	.
Verrucomicrobia	3	3

^a^“Half” indicates that the configuration required at least half the species of the group (e.g., 8 for Actinobacteria).

^b^A dot indicates that a configuration was never inferred for this group because of insufficient sampling.

### Analysis through “Configurations”

**Fig. 1.**
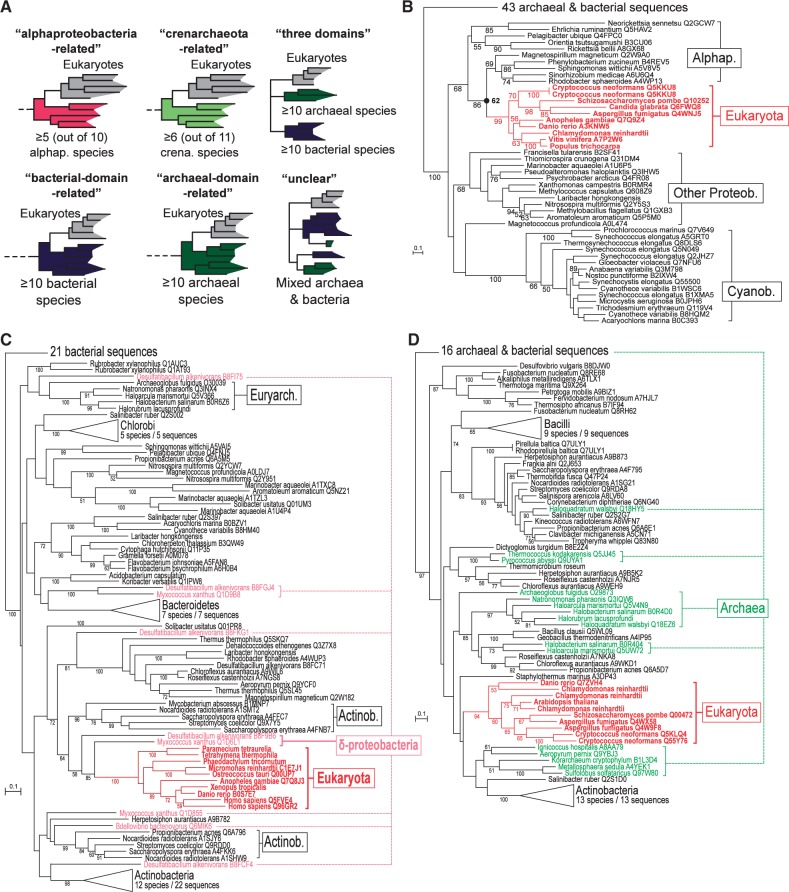
Gene trees were examined by means of configurations. (*A*) Schematic diagrams of six archetypal configurations. (*B–D*) Examples. The taxonomic sampling is always that of table 1. The numbers on branches represent nonparametric bootstrap supports (values below 50% are not shown). (*B*) ML tree of the hydroxybenzoate polyprenyltransferase (COQ2) LECA clade, which was annotated as “alphaproteobacteria-related.” The node at the base of the stem of eukaryotes, which NBS support was 62%, is marked by a black circle. (*C*) ML tree of the “long-chain acyl-CoA ligase” LECA clade. The sister group of eukaryotes consisted of an isolated *M. xanthus* sequence, which is likely the result of a recent HGT as most of the seven other Deltaproteobacteria do not encode related sequences. Therefore, this LECA clade was annotated as bacterial-domain-related (related to bacteria, but not to any phylum in particular). (*D*) ML tree of the “4-nitrophenylphosphatase” LECA clade, annotated as unclear because archaeal (in green) and bacterial (in black) sequences were mixed.

The trees were extremely heterogeneous in terms of species content, number of paralogs per genome, branching patterns, as well as in terms of branch length and bootstrap support distributions among branches (e.g., fig. 1*B*–*D*). This extensive diversity made the definition of standardized analysis principles very challenging. One possibility was to consider that the closest relatives of a LECA clade are the organisms constituting its sister group. This principle is intuitive, but clearly too naive. Even though it worked well in some cases (e.g., fig. 1*B*), it often led to questionable conclusions, owing to HGTs among prokaryotes and the incompleteness of sampling (e.g., fig. 1*C* and Discussion). Therefore, to establish relationships between eukaryotes and prokaryotic groups, we relied on extended topological criteria we refer to as configurations. Configurations take into account the taxonomic identity of the sister group of eukaryotes and that of the neighboring groups as well as, most importantly, the taxonomic representativeness of these groups, according to a system of thresholds (fig. 1*A*, table 1, and Materials and Methods).

### Archaeal-Bacterial Mosaicism

**Fig. 2.**
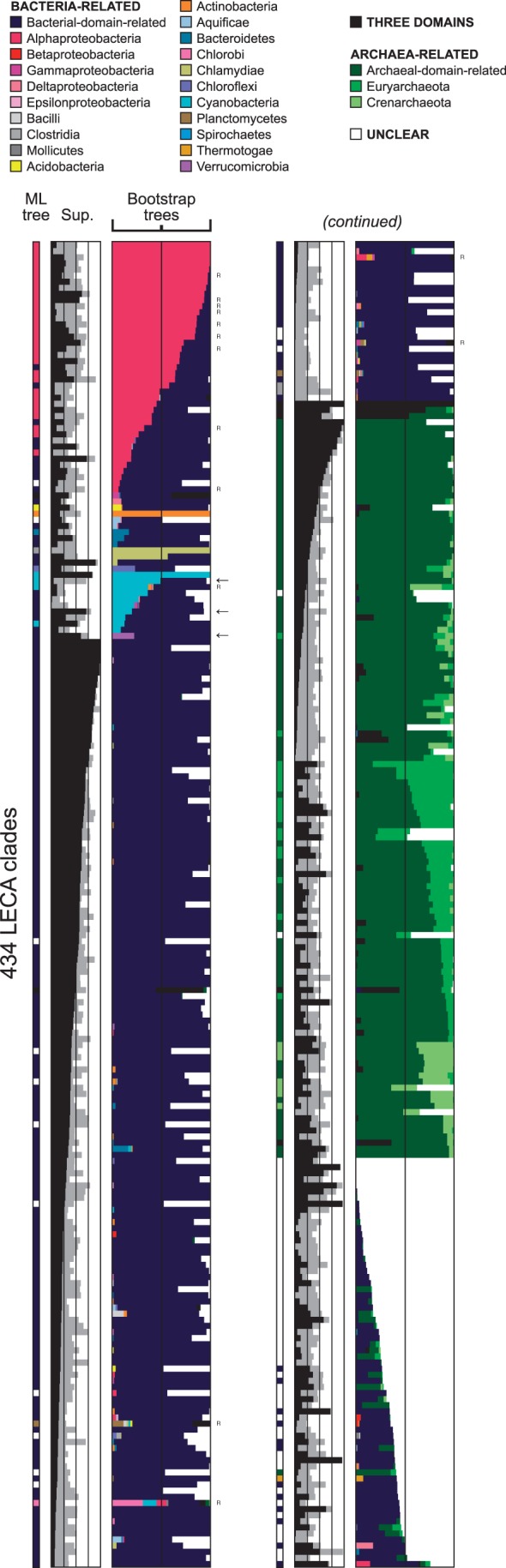
Inferred prokaryotic origins of eukaryotic genes. Each row represents 1 of 434 LECA clades and reports, from left to right, the configuration of its ML tree (the color code is given by the legend, top), the local topological support (“Sup.” column; NBS and SGS are in black and gray, respectively), and the configurations that appear in bootstrap trees. LECA clades are sorted by configurations and decreasing node support. A “R” letter on the right indicates that the gene is encoded in the mitochondrial genome in *R. americana*. Overall, 41 LECA clades were traceable to Alphaproteobacteria (pink), 24 to other bacterial phyla, among which 3 were so with high support values (arrows, and see Results), 177 to Bacteria though not to a particular taxonomic group (bacterial-domain-related, deep blue), while three appeared in the three-domain (3D) configuration (black), 117 were related to Archaea (green), and 71 were of unclear origin (white).

For each of the 434 supported LECA clades, we determined the configuration of the ML tree and those of all bootstrap trees. Results are summarized in figure 2. They were highly robust to alignment and tree reconstruction methods (supplementary fig. S1, Supplementary Material online). Based on the “most frequent configuration among bootstrap trees” criterion, 243 LECA clades appeared as being of bacterial origin, 121 as being of archaeal origin, while the “three-domain” configuration, with Archaea, Bacteria, and Eukarya all monophyletic, was recovered in only three cases. Finally, the “unclear” configuration, corresponding to tangled histories in which Archaea and Bacteria appeared mixed (e.g., fig. 1*D*), occurred for 67 LECA clades.

### Relations of Eukaryotes to Bacterial Phyla

To discriminate between the different hypotheses for the origin of eukaryotes, which predict contributions from different organisms, we performed an in-depth phylogenetic analysis for each of the 243 bacteria-related LECA clades. As expected, given that mitochondria are derived from Alphaproteobacteria, a substantial number of LECA clades (24) were found to be associated with representative alphaproteobacterial sequences in at least 50% of their bootstrap trees (fig. 2), and 17 more were so at lower thresholds. Three of these genes were alphaproteobacteria-specific but most were widely distributed in Bacteria. Almost all of them (38 out of 41) were involved in core mitochondrial functions such as protein processing (translation, chaperones), respiration (tricarboxylic acid cycle, oxidative phosphorylation, ATP synthase), and Fe-S cluster biosynthesis.

In addition, our analysis identified 24 LECA clades that might be related to bacterial phyla other than alphaproteobacteria (fig. 2). These clades were further investigated for possible sampling and clustering artifacts (see Materials and Methods), and the ML-tree bootstrap supports were considered in the classical way. For three of them, the proposed origin was well supported (univocal phylogeny and more than 75% bootstrap support at key branches). They were related to Cyanobacteria (two LECA clades) and Verrucomicrobiae (one LECA clade). For 19 clades, the proposed origin lacked bootstrap support. For the last two clades, it proved misguided because the taxonomic distributions of these genes in prokaryotes were particularly patchy and were initially not properly sampled (e.g., supplementary fig. S2, Supplementary Material online).

In total, we identified 41 LECA clades as reliably traceable to alphaproteobacteria and 3 to other bacterial groups. But the remaining 198 bacteria-related LECA clades, although clearly related to Bacteria, could not be traced back to a particular phylum. These cases were labeled “bacterial-domain-related.” They could be explained in several ways. According to the thermoreduction hypothesis (Forterre 2011), which is based on a three-domain tree of life rooted on the bacterial branch, these LECA clades were inherited from LUCA and appear related to Bacteria because of losses in Archaea: they are the sister group of Bacteria, rather than deriving from them. Consequently, these genes should also have been present in the last bacterial common ancestor (LBCA). This was in many cases questionable. For 100 of the 198 bacterial-domain-related LECA clades, fewer than half of the bacterial genomes encoded a homolog. In addition, presence–absence and branching patterns indicated that many duplications, transfers, and losses of these genes occurred. Their presence in the LBCA was therefore dubious. Furthermore, 41 of the 98 remaining genes could be rooted, thanks to the presence of Archaea or deep paralogy. In all these trees, the LECA clade did not branch at the root but appeared to derive from Bacteria. The “archaeal losses” explanation was thus not supported.

Alternatively, bacterial-domain-related LECA clades may actually derive from Bacteria, but be untraceable to a particular taxonomic group because of HGTs among prokaryotes or lack of phylogenetic signal (or a combination of both). These two causes can be distinguished by examining the level of statistical support. Remarkably, some bacterial-domain-related LECA clades had well-supported relations with particular prokaryotic sequences. For 23 of them, the branching point of eukaryotes among prokaryotes had a node bootstrap support (NBS; see Materials and Methods) greater than 75%. NBS is directly comparable with the classical bootstrap branch bootstrap support: the support values of the branches surrounding a node are always higher than the NBS of this node (e.g., fig. 1*B*). Thus, for these 23 LECA clades, significant support existed. Strong evidence for HGTs among prokaryotes was found, as the sister group of eukaryotes was composed either of a few sequences from unrelated organisms or of an abnormally isolated sequence such as in figure 1*C*.

**Fig. 3.**
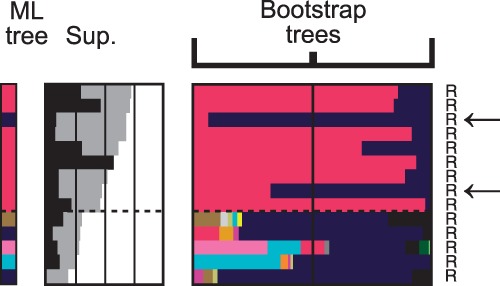
Ability of our approach to recover the alphaproteobacterial origin of mitochondrially encoded genes. Fourteen LECA clades (among 434) corresponded to genes that are encoded in the mitochondrial genome in *R. americana*. Figure is to be read like figure 2, except that LECA clades are sorted by decreasing (SGS, gray) support values. LECA clades having SGS values higher than 45% (dashed line) could be traced to Alphaproteobacteria, but those with lower supports could not, due to a lack of phylogenetic signal. For the third and eighth LECA clades from top (arrows), association with Alphaproteobacteria was weaker because of HGTs from Alphaproteobacteria to *Magnetococcus marinus* and Gammaproteobacteria, respectively.

However, relying on NBS is conservative. A high NBS at the base of a LECA clade guarantees the existence of signal, but a low one does not exclude high branch support values (fig. 1*B* and supplementary fig. S3, Supplementary Material online). As a matter of fact, the median NBS for the 41 LECA clades traceable to Alphaproteobacteria was only 24%. We thus designed a relaxed measure of support we refer to as “sister-group stability” (SGS; see Materials and Methods). We used the mitochondrion-encoded genes of *Reclinomonas americana* (which has one of the largest known mitochondrial genomes [Burger et al. 2013]) to calibrate this measure. The expected alphaproteobacterial origin was recovered for all genes with SGS above 45%, while it could not be so for genes with weaker support values (fig. 3, and see Materials and Methods). Retaining this 45% SGS threshold, 133 out of the 198 bacterial-domain-related LECA genes should be regarded as being somewhat supported, and our inability to determine their precise origin should be attributed to HGTs rather than to lack of signal. This, in addition to the fact that unresolved trees may also contain HGTs, and that many genes were taxonomically patchily distributed (supplementary fig. S1, Supplementary Material online), suggested that the primary cause for bacterial-domain-related annotations was HGT among prokaryotes.

### Relationship of Eukarya to Archaea

One important question regarding the relationship between Eukarya and Archaea is whether the latter are monophyletic or paraphyletic due to the branching position of the former, that is, whether the three domains are independent or not. Importantly, to assess this problem, only the genes that are widely present in Archaea, Bacteria, and Eukarya and were vertically inherited from LUCA are relevant. We therefore focused on clusters that were universal or nearly so (defined as containing representatives for at least 90% of species for both Archaea and Bacteria), and for which no clear evidence for HGTs was apparent. We also excluded bacteria-related LECA clades (e.g., mitochondrial proteins). These filters left 28 LECA clades (out of 434), most of which are involved in translation and have been used in other data sets of “universal genes,” for instance, those of Guy and Ettema (2011) or Williams et al. (2012) (supplementary table S1, Supplementary Material online).

**Fig. 4.**
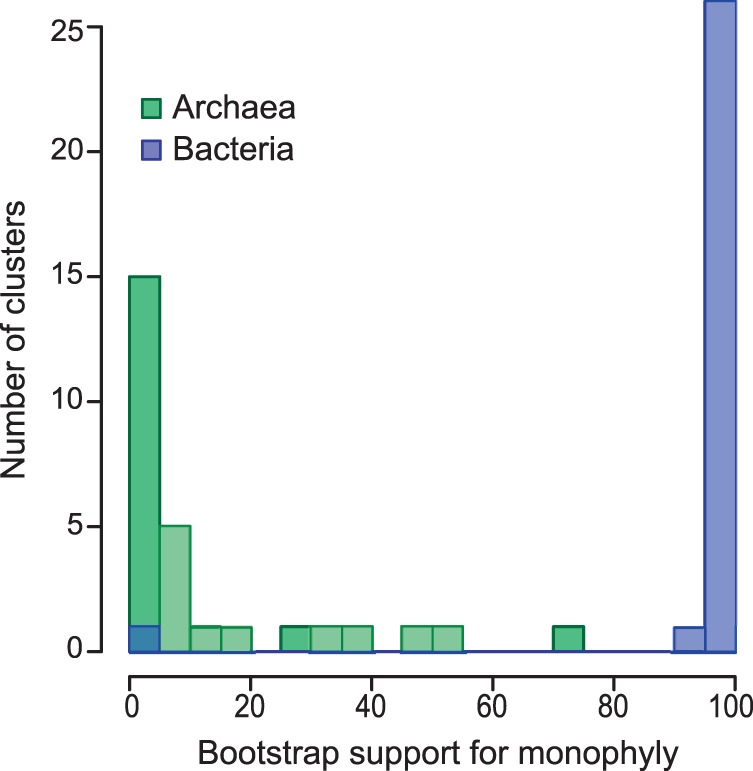
The missing support for the monophyly of Archaea. Histogram of bootstrap supports for the monophyly of Archaea and Bacteria in 28 nearly universal clusters of homologs. Although the monophyly of Bacteria was strongly recovered, that of Archaea was not, illustrating the fragility of the archaeal “domain” and the intimate relationship between Eukarya and Archaea.

In all 28 ML trees but one (ribosomal protein L23, which is very short), the monophyly of Bacteria was very strongly supported (fig. 4, mean bootstrap support: 95%). In contrast, the monophyly of Archaea was observed in only four ML trees, and accordingly there was no support for it (fig. 4, mean bootstrap support: 13%). Although it is tempting to take this result as evidence against the monophyly of Archaea, this is not the only possible interpretation. Upon closer inspection, we found that for many LECA clades the three-domain topology and the best paraphyletic-Archaea topology were equivalent: the likelihood difference between them was smaller than the default RAxML optimization error, meaning that they just could not be distinguished by standard means. It is also important to point out that there are many more possible topologies with Eukarya within Archaea (“paraphyletic–Archaea”) than three-domain ones. Paraphyletic–Archaea topologies thus likely comprise the bulk of the topologies that are almost as good as the true ML one. Hence, the high frequency of paraphyletic-Archaea topologies for near-universal genes may be the consequence of stochastic effects. Nevertheless, the ambiguity of the Eukarya–Archaea relationship contrasts sharply with the clear monophyly of Bacteria. The relationship between the three domains is markedly asymmetric; Archaea and Eukarya being much more intimately related to each other than they are to Bacteria. These results exclude a very distinct Archaeal domain and conversely support that Eukarya branch within Archaea or possibly close to them.

A second question is whether eukaryotes could be related to a particular archaeal lineage, such as methanogens or Thermoplasmatales. On this question, all of the 121 genes common to Archaea and Eukarya can be informative, notwithstanding the absence of bacterial homologs. Reviewing the trees, we found that the monophyly of archaeal orders was generally well supported, indicating that phylogenetic signal was present. Eukaryotes were not associated to any of them. A few markers recovered the monophyly of Crenarchaeota or that of Euryarchaeota with >80% bootstrap support (independently of the branching position of eukaryotes). These markers, which we regard as the most phylogenetically informative, placed eukaryotes outside of Crenarchaeota and of Euryarchaeota. Nevertheless, the branching order between Eukarya, Crenarchaeota, Euryarchaeota, Thaumarchaeota, and Korarchaeota remained unresolved. Overall, these analyses support that Eukarya branch deep within Archaea or close to their root if they are their sister group.

### Functions of Archaea- and Bacteria-Related Genes

KEGG groups of “orthologs” were used as a reference to map LECA clades on a functional ontology (see Materials and Methods and supplementary fig. S4, Supplementary Material online). As expected, systems such as the replication apparatus (e.g., replication factor C, MCM paralogs, ribonuclease H2), transcription complexes (e.g., RNA polymerases and nucleolar and spliceosomal complexes), and cytosplasmic protein processing (including the ribosome, translation factors, signal recognition particle, Sec61α, signal peptidase, methionine aminopeptidase, protein kinases and phosphatases, proteasome) were archaea-related. Mitochondrial protein processing genes were alphaproteobacteria-related, although some of them appeared as just bacterial-domain-related because of lack of signal. Intriguingly, one gene involved in mitochondrial RNA processing (PNPT1) was verrucomicrobiae-related. Few genes broke the “informational systems are archaea-related” rule. These include the SKI2/DOB1 family of accessory exosome subunits, and the MSH3 and NTG2 genes, which are involved in DNA repair.

Metabolism was overwhelmingly bacteria-related. Indeed, only a handful of metabolic genes were archaea-related (e.g., CTP synthase) while most of the 242 LECA clades of bacterial origin were involved in metabolism. Cellular respiration (tricarboxylic acid cycle, oxydative phosphorylation and its assembly factors, F-ATPase) was very strongly recovered as alphaproteobacteria-related. The Fe-S cluster assembly scaffold protein NifU was also alphaproteobacteria-related. Genes in other metabolic pathways were just bacteria-related, though a few isolated enzymes could be linked to alphaproteobacteria (aminomethyltransferase, LEU1, dihydroorotate dehydrogenase) or cyanobacteria (glutamate-5-kinase, decaprenyl-diphosphate synthase).

Lastly, we identified a few membrane transporters, which were either related to Bacteria in general or of unclear origin.

## Discussion

### Relevance of HOGENOM Clusters

We used phylogenomics methods to identify a large set of ancestral eukaryotic genes and investigate their relationships with their prokaryotic homologs. A fundamental step of all phylogenomics studies is the definition of sets of homologous sequences on which downstream analyses rely. Diverse strategies can be used to build such sets, including ones based on direct Blast (or profile-based) searches seeded with the species of interest (“centered” or “ingroup” strategies; Esser et al. 2004, 2007; Gabaldón and Huynen 2007; Cotton and McInerney 2010; Brindefalk et al. 2011; Thiergart et al. 2012), and ones that use an algorithm to extract families of homologous sequences from an all-vs.-all Blast matrix without a reference point (“decentralized” strategies; Tatusov 1997; Van Dongen 2000; Robbertse et al. 2011; Miele et al. 2012). In the present study, we used the clusters of homologs provided by the HOGENOM database, which are built in a decentralized manner (Penel et al. 2009; Miele et al. 2012).

Although the results produced by these strategies may be different, no systematic comparison has been performed yet and no objective indicators of strengths and flaws exist. Several lines of evidence indicate that the HOGENOM clusters are a sensible option. First, our attempts to enlarge clusters with new homologs, using HMM profiles seeded with the cluster’s sequences, yielded essentially sequences that were more distantly related to all of the seeds than seeds were to each other. HOGENOM clusters are therefore reliable and evolutionarily coherent sets. Second, we investigated the ability of our approach to recruit the 67 genes encoded by the mitochondrial genome of *R. americana*, which are all thought to have had ancestors in LECA. Using similarity searches, we could map 48 of these genes to a HOGENOM cluster, of which 25 could also be associated to one of our strictly defined LECA clades (see Materials and Methods). By comparison, approaches centered on *R. americana* (Esser et al. 2004, 2007; Brindefalk et al. 2011) or alphaproteobacteria (Gabaldón and Huynen 2007) included 42–55 *R. americana* genes, whereas another study based on decentralized clustering included only 20 (Thrash et al. 2011). The sensitivity of our methods on this test set was thus slightly reduced in comparison with centered approaches. Nevertheless, HOGENOM clusters have the advantage of being based on a formal implementation of the concept of a family of homologs (Miele et al. 2012). This implies that they are independent of our specific question, which reduces the risk that our conclusions could have been driven by preconceptions and facilitates their reproduction and assessment by third-parties.

### Polyphyly of Eukaryotic Sequences and Search for LECA Clades

As we searched for eukaryotic genes acquired from prokaryotes, the first step was to consider how frequently were eukaryotic sequences monophyletic regarding prokaryotic sequences from the same HOGENOM cluster. The HOGENOM clustering procedure does not consider taxonomy and is thus agnostic on this problem. We found that eukaryotic sequences were polyphyletic in 70% of the clusters. This is substantially more than the 20% figure recently reported by Thiergart et al. (2012). This divergence could be due, first, to a difference of sampling, as Thiergart et al. did not consider protist sequences, which may be particularly subject to HGT and/or artifacts such as long branch attraction. It is also possible that the two-step clustering procedure they used (eukaryotic sequences were clustered first, then prokaryotic sequences were added) may not have clustered as many distantly related eukaryotic sequences as in the HOGENOM procedure. Widespread existence of polyphyly is nevertheless expected because 1) for many proteins, such as those of the translation apparatus, eukaryotes have both archaea-related and bacteria-related copies, 2) plant genomes include genes of chloroplastic origin that branch with Cyanobacteria, 3) occasional prokaryote-to-eukaryote HGTs have occurred after the diversification of eukaryotes (Keeling and Palmer 2008; Marcet-Houben and Gabaldón 2010; Alsmark et al. 2013), and 4) lack of signal and/or artifacts that may prevent the monophyly of eukaryotes.

For these reasons, eukaryotic sequences from the same cluster of homologs should not be considered to be monophyletic a priori. For all clusters, we identified all clades of eukaryotic sequences and treated them as of putatively distinct origins. A cluster was inferred to trace back to LECA on the basis of the presence of at least two groups out of Plantae, Unikonts, and Chromalveolates plus Kinetoplastids. This design is similar to those used by Makarova (2005) and Thiergart et al. (2012), except that the criterion of the former (Makarova 2005) was more permissive (notably, it was met for opisthokont-specific genes) and the criterion of the latter (Thiergart et al. 2012) did not consider protists. It must be noted that, by any means, inferences of ancestrality in eukaryotes can only be rough because 1) the tree of eukaryotes (Hampl et al. 2009; Zhao et al. 2012) and its root (Roger and Simpson 2009; Rogozin et al. 2009; Cavalier-Smith 2010a; Derelle and Lang 2012) are debated, 2) the number of available protist genomes is limited, and 3) the amount of HGT among eukaryotes, especially protists, is unclear (Keeling and Palmer 2008; Hampl et al. 2011; Burki et al. 2012).

Eventually, 554 LECA-traceable clades with prokaryotic homologs were inferred, representing 777 and 546 human and yeast genes, respectively. Previous studies reported figures of 850 yeast genes (Esser et al. 2004), 203–842 at least (depending on the criteria used; Gabaldón and Huynen 2007), 386–415 at best (Pisani et al. 2007), 980 (Yutin et al. 2008), 2,460 yeast genes (Cotton and McInerney 2010), and 571 (Thiergart et al. 2012). The overall sensitivity achieved using HOGENOM clusters and stringent phylogenetic criteria was thus comparable with that obtained by other methods, except for the very permissive one used by Cotton and McInerney (2010).

### Eukarya and Archaea Are Intimately Related

We then investigated the relationships of all LECA clades with high-rank prokaryotic taxonomic groups. About one-third of them appeared archaea-related and two-thirds appeared bacteria-related (fig. 2). This is in agreement with previous observations of the apparent mosaicism of eukaryotes, which have reported similar archaeal-over-bacterial gene ratios (Esser et al. 2004; Yutin et al. 2008; Thiergart et al. 2012). The strong enrichment for informational and metabolic functions among archaea-related and bacteria-related genes, respectively (Koonin 2010), was also recovered.

Regarding the archaea-related eukaryotic genes, our results were dominated by two trends. First, in near-universal gene phylogenies, the monophyly of Bacteria was prominent but the monophyly of Archaea (relative to Eukarya) was not supported at all (fig. 4), suggesting a very close relationship between Eukarya and Archaea. Nevertheless, our analyses did not support a specific branching order for archaeal phyla or a particular position of Eukarya relative to them.

Hence, our results are compatible with the views that Eukarya are a sister group of Thaumarchaeota–Aigarchaeota, Crenarchaeota and/or Korarchaeota, as supported by the latest dedicated studies (Guy and Ettema 2011; Kelly et al. 2011; Williams et al. 2012; Lasek-Nesselquist and Gogarten 2013). They are also, in principle, compatible with the three-domain view (in which Eukarya are the sister group of all Archaea) (Brown et al. 2001; Ciccarelli et al. 2006), though they would, in this case, support a short archaeal stem branch. Remarkably, several hypotheses strictly depend on the three-domain view and state that the last archaeal common ancestor (LACA) was very different from the one of Archaea and Eukarya (LAECA) (Cavalier-Smith 2010b; Forterre 2011). These large differences would have evolved along the archaeal stem branch. These hypotheses seem to conflict with currently available phylogenetic results.

Second, among all the archaea-related LECA clades we identified, none is soundly related to any particular archaeal lineage when statistical support and HGT are considered. Phylogenetic signal was strong at the order level, so our results go against a specific relationship between Eukarya and *Ignicoccus* (Küper et al. 2010; Godde 2012), *Pyrococcus* (Horiike et al. 2004), or *Thermoplasma* (Margulis et al. 2000). The most informative markers shared between Archaea and Eukarya (but absent from Bacteria) consistently supported a deep branching of Eukarya relative to archaeal phyla and conversely excluded that Eukarya emerged from within Crenarchaeota or Euryarchaeota. This is also in agreement with concatenation studies (Guy and Ettema 2011; Williams et al. 2012). Importantly, a deep branching position disputes that eukaryotic ancestors could have been methanogenic, as proposed by the “hydrogen” and “syntrophic” hypotheses (Martin and Müller 1998; Lopez-Garcia and Moreira 2006), because methanogenesis is thought to have evolved only once, in Euryarchaeota, after the divergence of Thermococcales, and have not been transferred to other groups (Gribaldo and Brochier-Armanet 2006).

### A New Picture of the Origins of “Bacteria-Related” Eukaryotic Genes

We found that bacteria-related eukaryotic genes could be mainly divided into two sets: genes involved in core mitochondrial functions and related to Alphaproteobacteria, which are clear EGTs, and genes for which it is not possible to determine a precise origin within Bacteria, usually because of the piling of HGT and gene losses in bacteria (before and/or after the origin of eukaryotes) but sometimes because of a lack of phylogenetic signal.

**Fig. 5.**
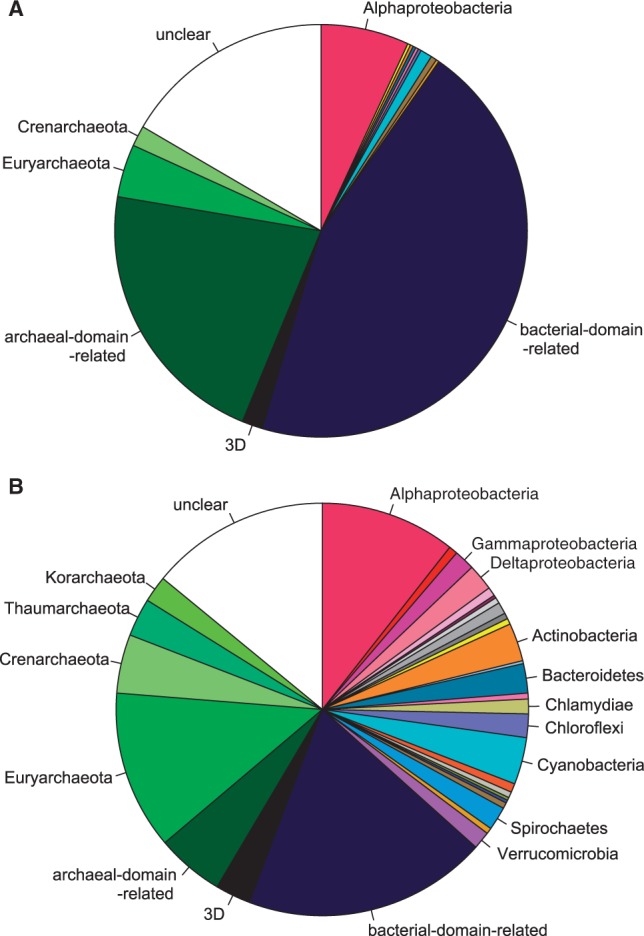
The impact of configurations on the determination of the origins of ancestral eukaryotic genes. The diagrams represent the origins of 434 LECA clades as inferred from their ML trees using (*A*) configurations or (*B*) the simpler but naive sister-clade-identity criterion. The colors correspond to the legend given in figure 2. Labels corresponding to fewer than five LECA clades were omitted. The sister-clade-identity criterion was overconfident regarding vertical inheritance and generated many spurious annotations. In contrast, configurations conservatively interpret the phylogenies where peculiar taxonomic distributions suggest HGTs, like in figure 1*C*. See supplementary figure S5, Supplementary Material online, for a more detailed comparison.

This division into two sets sharply contrasts with earlier studies (Pisani et al. 2007; Saruhashi et al. 2008; Koonin 2010; Szklarczyk and Huynen 2010; Thiergart et al. 2012), where eukaryotic genes appeared related to diverse bacterial phyla. The discrepancy arises from the use of taxonomy-aware criteria when inferring eukaryotic gene origins. Indeed, if we disregarded configurations and opted for a naive sister-group-identity criterion, we observed a pattern of diverse origins very similar to the one reported by previous studies (fig. 5).

The simpler criterion is actually unsuitable to assess the origins of eukaryotic genes, because it does not recognize the importance of HGT and gene loss dynamics nor that of lack of signal. For instance, in figure 1*C*, the closest relatives of eukaryotes are sequences from *Myxococcus xanthus* and *Desulfatibacillum alkenivorans*, two Deltaproteobacteria. Yet, given that this tree was built using a data set comprising eight representative deltaproteobacterial genomes (table 1), it is unlikely that these sequences were inherited vertically from a billion-year-old deltaproteobacterial ancestor and lost in other Deltaproteobacteria. They are more probably recent HGTs from an unsampled lineage. It is thus unclear whether the eukaryotic sequences derive from Deltaproteobacteria. Conversely, figure 1*B* shows a tree in which eukaryotes branch within a group of alphaproteobacterial sequences that represent all ten sampled alphaproteobacterial genomes. In that case, the most likely scenario is that this gene was ancestral to Alphaproteobacteria and transferred to eukaryotes by EGT from the mitochondrion.

Hence, the “diverse origins” pattern is due to the use of a too simple criterion. Some authors tempered this pattern a posteriori (Thiergart et al. 2012), but this meant giving up on effectively disentangling the several possible underlying causes for it. In contrast, we addressed the prevalence of HGTs and gene loss in prokaryotes at the methodological level using taxonomy-aware criteria (fig. 1*A*) and a balanced selection of prokaryotic genomes (table 1). This, in addition to our consideration of phylogenetic support throughout the analysis, allowed us to reveal and quantify the roles of EGT, HGT from bacteria into the eukaryotic stem branch, HGT among bacteria, and lack of signal. For these reasons, the picture we report is more accurate and reliable than the “diverse origins” one.

### No Phylogenetic Support for Ternary Scenarios

One major and new result brought about by our approach is that, while the alphaproteobacterial nature of mitochondria is very clear, there is no phylogenetic evidence for eukaryotes to have similarly inherited genes from another bacterial lineage. This observation is of special interest for ternary hypotheses, which advocate that bacteria-related eukaryotic genes descend in part from the ancestor of mitochondria and in part from another bacterial lineage. We found absolutely no traces in support of such an admixture. This lack of evidence questions the relevance of these hypotheses, especially as they suppose the most unconventional cellular mechanisms (Cavalier-Smith 2010b; Forterre 2011).

The early-mitochondria hypotheses (Martin and Müller 1998; Vellai et al. 1998; Searcy 2003) advocate that the genes of the proto-mitochondrion massively replaced those of the host through EGT so that bacteria-related eukaryotic genes derive from an alphaproteobacterial genome. This origin is clear for genes involved in core mitochondrial functions such as protein processing and respiration. However, bacteria-related genes functioning elsewhere in the cell do not link to Alphaproteobacteria in particular. Thus, there is no evidence that those genes were acquired as a result of a massive genetic transfer subsequent to the mitochondrial endosymbiosis. Nevertheless, early-mitochondria hypotheses cannot be excluded either, because they can be made compatible with these results by hypothesizing that bacteria-related eukaryotic genes actually come from an alphaproteobacterial genome, but that these origins are masked by recent and/or ancient HGTs among prokaryotes (Martin 1999; Esser et al. 2007).

Finally, the “slow-drip” hypothesis proposes that bacteria-related eukaryotic genes unrelated to Alphaproteobacteria were acquired by stem eukaryotic ancestors by HGT from diverse bacteria and actually have no links with the mitochondrial endosymbiosis. This hypothesis further suggests that those transfers occurred through prokaryotic-like HGT mechanisms (in contrast with the “you-are-what-you-eat” (Doolittle 1998) hypothesis, in which they are mediated by phagocytosis). The slow-drip scenario thus predicts that the bacteria-related, mitochondria-unrelated gene set should be enriched for genes that frequently transfer among prokaryotes. This implies that in most cases, the precise origin of bacteria-related eukaryotic genes should be blurred by HGT. This is what we observe. Hence the apparent phylogenomic patterns at the origin of eukaryotes can also be interpreted as the outcome of a slow-drip scenario.

## Conclusion

The mosaicism of the eukaryotic genome is challenging. We demonstrate why determining the evolutionary histories of its genes precisely is difficult and often impossible given currently available genomic data and phylogenetic methods. Nevertheless, our analysis establishes that there is no phylogenomic support in favor of ternary hypotheses. In addition, we present evidence that single-gene phylogenies collectively exclude a close relationship between Eukarya and Crenarchaeota or Euryarchaeota and support that Eukarya branch close to Archaea or basally within them. This is at odds, in particular, with hypotheses in which eukaryotes derive from methanogens. Finally, we show that the slow-drip hypothesis and some early-mitochondria hypotheses are compatible with current genomic data under certain assumptions.

Further progress on the question of the origin of eukaryotes is expected to arise from new genome sequences of undersampled archaeal and eukaryotic lineages, better methods for reconstructing taxon-rich single-gene phylogenies, and better knowledge of the biological diversity of Bacteria and Archaea.

## Materials and Methods

### Identification of LECA Clades

The HOGENOM (v5) database includes all proteins from 64 eukaryotic, 62 archaeal, and 820 bacterial complete genomes, and provides precomputed clusters of homologs based on all-vs- all Blasts and transitive homology bonds (Penel et al. 2009; Miele et al. 2012). HOGENOM clusters containing two groups out of Opisthokonts, Plantae, and Chromalveolates, and at least one prokaryotic phylum, were retrieved, along with their ML trees. Because no tree was available for the 20 largest clusters (>2,000 sequences), they were not analyzed further. All monophyletic clades of eukaryotic sequences were extracted by means of custom tree-parsing algorithms implemented using the Bio++ (Dutheil et al. 2006) C++ library. Eukaryotic clades were inferred to trace back to LECA if they contained sequences from at least 1) two Unikont species and two Plantae, 2) two Unikonts and two Chromalveolates, or 3) two Plantae, two Chromalveolates and one kinetoplastid. Because recent eukaryotes-to-prokaryotes HGTs may confuse this strategy by making eukaryotes appear paraphyletic, all trees were manually inspected before eukaryotic clades were extracted, and isolated prokaryotic sequences branching within a group of diverse eukaryotes were removed.

### Sampling of Sequences in LECA Clades

For each LECA clade, we selected sets of representative sequences while trying to exclude the sequences with the longest branches. An ML tree of the clade’s sequences was built using MUSCLE (Edgar 2004), Gblocks (Talavera and Castresana 2007) and FastTree (Price et al. 2010) and then rooted using the least-squares criterion (implemented in Bio++). Leaves were pruned iteratively until ten sequences were left, removing at each round the sequence that was the furthest from the root nodewise and the furthest branch-lengthwise among draws (implemented in Bio++). The selections were then manually inspected and adjusted when relevant. The sets of sequences gathered this way represented the sequence diversity and not necessarily the taxonomical one.

### Sampling of Bacterial and Archaeal Genomes

All analyses except the identification of LECA clades were performed using the same subset of 183 representative archaeal and bacterial genomes. These genomes were chosen as follows. In Archaea, one genome was sampled in each represented genus, except *Nanoarchaeum equitans* (which was not included because of its high evolutionary rate and uncertain phylogenetic position), for a total of 39 genomes. In Bacteria, up to 15 genomes were sampled for each phylum, except for Proteobacteria and Firmicutes, which were sampled classwise. Representatives were selected according to a reference species phylogeny (Wu et al. 2009). For bacterial phyla for which genomes were available for less than 15 genera, one genome was randomly sampled in each genus. Overall, 144 bacterial genomes were included.

### Phylogenetic Inferences

Trees and results presented in figures were obtained using Probcons (default parameters; Do et al. 2005), BMGE (BLOSSUM30 matrix; Criscuolo and Gribaldo 2010), and RAxML (CAT rates, LG model, 100 nonparametric bootstrap replicates) (Stamatakis 2006). Analyses were replicated using MAFFT (E-INS-i mode; Katoh and Toh 2008), guidance (default parameters, working with MAFFT-E-INS-i; Penn et al. 2010), Phylobayes (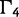 rates, LG model, with fixed equilibrium frequencies; Le, Gascuel, et al. 2008), and PhyML-structure (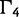 rates, UL3 model; Le, Lartillot, et al. 2008) (supplementary fig. S1, Supplementary Material online). Constrained (three-domain) reconstructions were performed using RAxML. Computations were run locally and on the IN2P3 cluster (http://www.in2p3.fr/, last accessed January 13, 2014) and lasted for about 20,000 CPU hours.

### Configurations

The configuration of every bootstrap and ML tree was determined as follows. A LECA clade was said to be related to a particular phylum (or class for Proteobacteria and Firmicutes) if it branched inside a clade of sequences of this phylum and that these sequences represented a number of species higher than the threshold given in table 1 (e.g., fig. 1*A*). Similarly, a LECA clade was said to be bacteria-related (respectively, archaea-related) if it branched inside a clade of bacterial (respectively, archaeal) sequences representing at least ten species (fig. 1*A*). A LECA clade that was bacteria-related (respectively archaea-related) but could not be related to a given phylum was labeled bacterial-domain-related (respectively, “archaeal-domain-related”). A tree was labeled three-domain if all the three domains were monophyletic and at least ten archaeal and ten bacterial species were represented (fig. 1*A*). A tree in which the LECA clade was neither bacteria-related, nor archaea-related, nor in a three-domain position (fig. 1*A*), was labeled unclear. Trees in which the representative sequences for a LECA clade were paraphyletic were labeled “paraphyletic” and discarded. The identification of configurations was implemented using Bio++. Source code is available upon request.

### Inspection of LECA Clades Putatively Related to Bacterial Groups Other than Alphaproteobacteria

The cases of these clades were investigated individually. First, their ML trees (built using 183 prokaryotic genomes) were compared with the ones built using the 882 prokaryotic genomes of HOGENOM (v5), to check that the smaller genome set allowed for a proper sampling of the sequence diversity, and to exclude oddities such as the one presented in supplementary figure S2, Supplementary Material online. In addition, the reliability of the HOGENOM clustering was checked by performing a HMMER 3.0 (Eddy 2011) search in the 183 complete proteomes, using as seed a MAFFT (default FFT-NS-2 mode) alignment of the cluster, and then verifying that the top hits were the cluster’s sequences. Finally, we reviewed the robustness of the scenarios suggested by the ML trees, considering the taxonomic distributions, potential HGTs, and bootstrap support values. An archive file containing the lists of species and genes, the alignments, and the trees used in this study is available at ftp://pbil.univ-lyon1.fr/pub/datasets/rochette/Rochette2014_origin_euks.tar.gz (37Mb).

### Support Measures

The classical phylogenetic support measure, the branch bootstrap support, cannot be used to characterize the branching position of a LECA clade among prokaryotic sequences because this position does not depend on one single branch. Two alternative support measures were used.

The NBS is defined as the percentage of bootstrap replicate trees in which this node (i.e., tripartition) occurs, which is equivalent to saying that the three branches (i.e., bipartitions) adjacent to this node cooccur. This support was computed in each tree for the node at the base of the stem of eukaryotes as it is the one that contains most information regarding their branching position among prokaryotes.

The SGS score measures the stability of the set of prokaryotic sequences in the sister group of a given LECA clade across bootstrap replicates. The sister group of eukaryotes here refers to the smallest of the two prokaryotic subtrees separated by the node at the base of eukaryotes. It is defined as

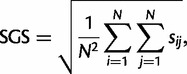

where *N* is the number of bootstrap trees (i.e., 100) and

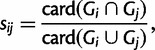

where *G_i_* and *G_j_* are the sets of leaves in the sister groups of eukaryotes in bootstrap trees *i* and *j,* respectively. When eukaryotes are paraphyletic in *i* or *j*, 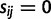. This score ranged from 0 (complete disjunction between sister groups in different replicates) to 1 (absolute stability of the sister group).

The SGS and NBS supports are related. By construction, the SGS score is at least as high as the NBS of the node at the base of the eukaryotic stem, which corresponds to

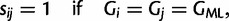

where *G*_ML_ is the sister group of eukaryotes in the ML tree of this LECA clade.

### Mitochondrion-Encoded Genes in R. americana

Because the nuclear genome of *R. americana* is not sequenced, this species is absent from HOGENOM. The 67 proteins encoded in its mitochondrial genome were retrieved from Uniprot (http://uniprot.org/, last accessed January 13, 2014) via the “AF007261” EMBL tag of the mitochondrial genome. They were mapped to HOGENOM clusters using Blast (Altschul et al. 1997) with a 30% identity threshold. Affiliation to a LECA clade was then inferred, for each sequence, by manual examination of an ML tree including the *R. americana* sequence in addition to the sequences of the cluster for 183 prokaryotic and 19 eukaryotic representative genomes and built using MAFFT (default FFT-NS-2 mode), BMGE, and FastTree (Price et al. 2010).

### Mapping of LECA Clades to KEGG Orthologs Groups

For each LECA clade, the Kyoto Encyclopedia of Genes and Genomes (KEGG) identifiers of the sequences of six model eukaryotes were retrieved from HOGENOM through their Uniprot identifiers. Their cards were retrieved from the KEGG website (http://genome.jp/kegg/, last accessed January 13, 2014) using GNU’s wget tool and the identifiers of the groups of homologs they belonged to (“K” identifiers) were extracted. In some cases, several HOGENOM clusters corresponded to a single KEGG group, due to a wider KEGG clustering, or conversely one HOGENOM cluster could point to several KEGG groups, due to the division of some gene families according to duplication–neofunctionalization events. The “KEGG Orthology” ontology (functional ontology of the groups of homologs) was obtained from the KEGG website.

## Supplementary Material

Supplementary figures S1–S5 and table S1 are available at *Molecular Biology and Evolution* online (http://www.mbe.oxfordjournals.org/).

Supplementary Data
