# Supplementary material for: Phylogenomic Test of the Hypotheses for the Evolutionary Origin of Eukaryotes
Source: Mol Biol Evol. 2014 Jan 7;31(4):832–45. doi: 10.1093/molbev/mst272 (PMC3969559; doi:10.1093/molbev/mst272)
Supplement: Supplementary Data [file supp_31_4_832__index.html]

Phylogenomic test of the hypotheses for the evolutionary origin of eukaryotes — Phylogenomic Test of the Hypotheses for the Evolutionary Origin of Eukaryotes — Phylogenomic Test of the Hypotheses for the Evolutionary Origin of Eukaryotes — Supplementary Data 

# Phylogenomic Test of the Hypotheses for the Evolutionary Origin of Eukaryotes

## Supplementary Data

files

**Files in this Data Supplement:**

- Supplementary Data - pdf file
