# Supplementary material for: Phylogenomic Test of the Hypotheses for the Evolutionary Origin of Eukaryotes
Source: Mol Biol Evol. 2014 Jan 7;31(4):832–45. doi: 10.1093/molbev/mst272 (PMC3969559; doi:10.1093/molbev/mst272)
Supplement: Supplementary Data [file supp_mst272_SI.pdf]

Supplementary figures

Figure legends

Figure S1:

Comparison of the annotations inferred on trees built with Probcons+BMGE+RAxML (left panel, same as article Fig. 2), Probcons+BMGE+Phylobayes (middle panel) or MAFFT-EINSI+Guidance+RAxML (right panel). The annotations obtained on the Probcons+BMGE+PhyML (UL3 model) maximum-likelihood trees are also reported (left panel, "UL3" column). The order of LECA clades is the same in all panels. LECA clade identifiers and definitions are reported on the right of each line. White lines in Phylobayes and MAFFT-Guidance panels correspond to LECA clades for which the support for the monophyly of eukaryotes was under 50% using those alternative approaches, or (for Phylobayes only) to LECA clades whose MCMC chains were stopped before convergence was achieved (i.e. maximum bipartition discrepancy <0.3). The correlation between all three approaches is excellent, except the topologies sampled with the Bayesian method are generally distributed more narrowly around the ML topology, as expected [1]. For each cluster, the species-presence profile is summarized on the left. Each column corresponds to a high-order prokaryotic group. A small colored circle indicates that a few species of the group are present ; a large colored circle indicates that more than half the species of the group are present (the thresholds given in article table 1 are used) ; a black ring indicates that a clade comprising at least half the species of the group exists in the ML tree of the cluster.

1. Douady CJ, Delsuc F, Boucher Y, Doolittle WF, Douzery EJP: **Comparison of Bayesian and Maximum Likelihood Bootstrap Measures of Phylogenetic Reliability**. *Mol Biol Evol* 2003, **20**:248–254.

Figure S2:

Phylogenies of the LECA gene HBG298928_1 ("Alpha/beta hydrolase fold protein") using 183 (left) or 882 (right) prokaryotic genomes. This figure illustrates one of the subtle issues caused by sampling. At first this gene was labeled "actinobacteria-related" on the basis of the 183-prokaryotic-genomes dataset, as all its homologs were actinobacterial. It then appeared using the 882-prokaryotic-genomes dataset that it was also present at low frequencies in at least three orders of gammaproteobacteria. In addition, depending on where the larger tree was rooted, the sister group of Eukarya was Actinobacteria, Gammaproteobacteria, or both. Thus an important part of the molecular history of this family of homologs was missed, what rendered the initial annotation questionable.

Figure S3:

Phylogeny of the LECA gene HBG487932_1 ("Cytochrome b"). This tree illustrates the limits of considering only the support of the node at the base of the stem of Eukaryotes. For this LECA clade, the NBS support was 7%, even though the tree is rather well resolved. In contrast, the SGS score was 68%.

Figure S4:

LECA clades displayed according to the first and second levels of the KEGG ORTHOLOGY ontology. Some LECA clades may appear in several categories.

Figure S5:

Comparison of the annotations of LECA clade positions based on the “reference” configurations (article Fig. 1), a “relaxed” criterion or a “naive” one. (A) Schematic diagrams for the criteria. The relaxed criterion was similar to the reference one except eukaryotes are allowed to branch as a sister group of the putatively related prokaryotic sequences (when the reference one required that they branched among them). With the naive criterion, a relationship was inferred whenever eukaryotes have a taxonomically homogeneous sister clade, even when this clade was made of only one sequence (e.g. main-text Fig. 1C). (B) Annotations obtained using the naive (left), relaxed (middle) or reference (right) criteria. The figure is to be read like main-text Fig. 2. The LECA clades appear in the same order in the three panels. The sorting is based on the left panel (note that the rows of the right panel are the same as those of article Fig. 2, but resorted).

The greatest difference was between the naive and relaxed criteria, that is when taxonomic representativeness criteria were introduced. Most often, the relaxed and reference criteria differed only quantitatively. The reference criterion was adopted because it was immune to rooting issues.

Interestingly, the ability of the method to detect putatively alphaproteobacterial genes varied little with the criterion used. Indeed, the number of candidate alphaproteobacteria-related LECA clades using the naive and reference criteria were respectively 46 ($\>$50\% of alphaproteobacteria-related bootstrap replicates) and 41 ($\>$5\% of alphaproteobacteria-related replicates representing more than 80\% of all replicates annotated to a specific taxonomic group). The reason for this result is that configurations were much more specific, so that even LECA clades for which the alphaproteobacteria-related configuration appeared in a few replicates could be considered candidates.

Figure S1

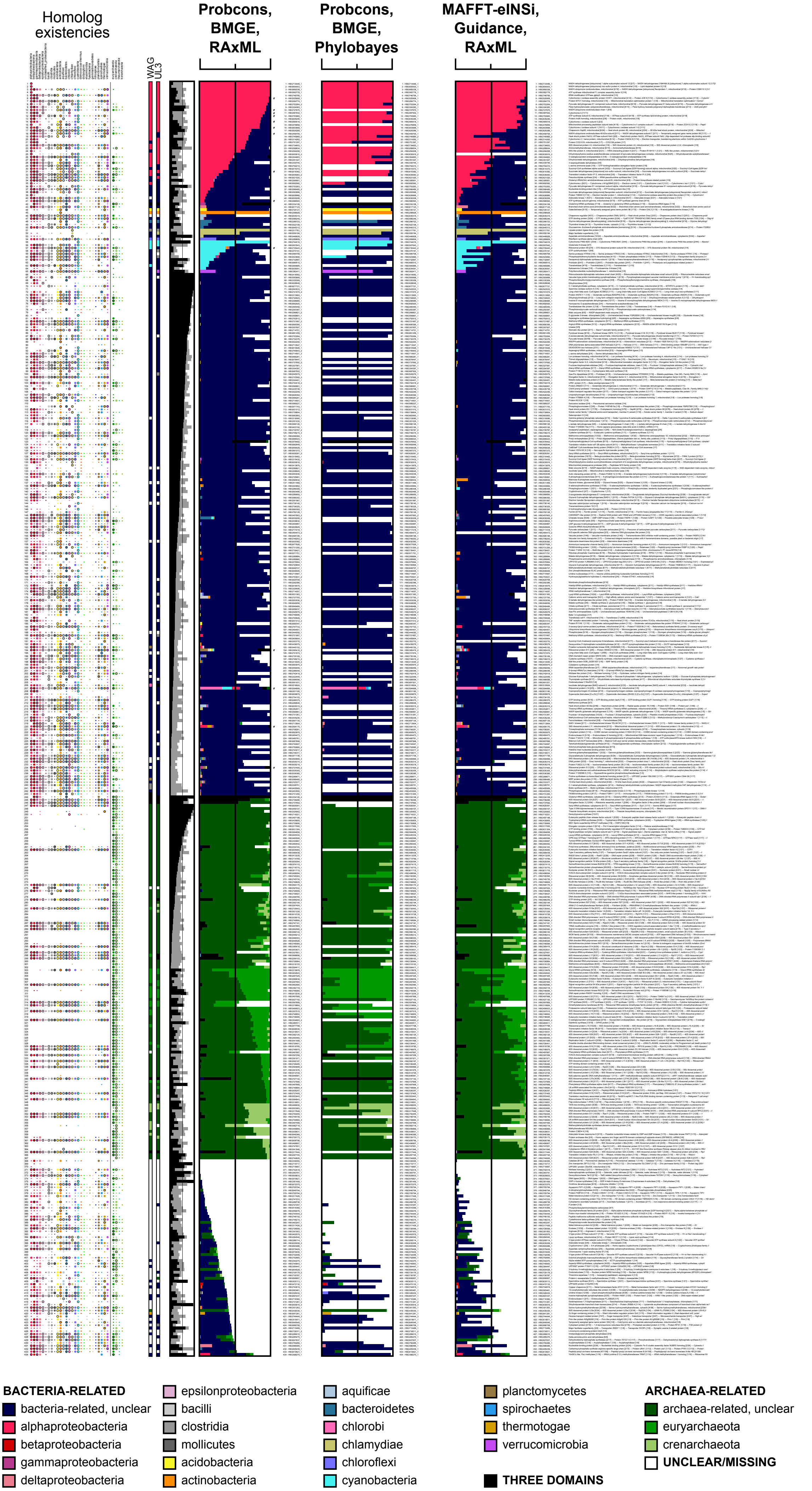
Homolog existencies
WAG
UL3
Probcons, BMGE, RAxML
Probcons, BMGE, Phylobayes
MAFFT-eINSi, Guidance, RAxML
BACTERIA-RELATED
bacteria-related, unclear
alphaproteobacteria
betaproteobacteria
gammaproteobacteria
deltaproteobacteria
epsilonproteobacteria
bacilli
clostridia
mollicutes
acidobacteria
actinobacteria
aquificae
bacteroidetes
chlorobi
chlamydiae
chloroflexi
cyanobacteria
planctomycetes
spirochaetes
thermotogae
verrucomicrobia
THREE DOMAINS
ARCHAEA-RELATED
archaea-related, unclear
euryarchaeota
crenarchaeota
UNCLEAR/MISSING

Figure S2

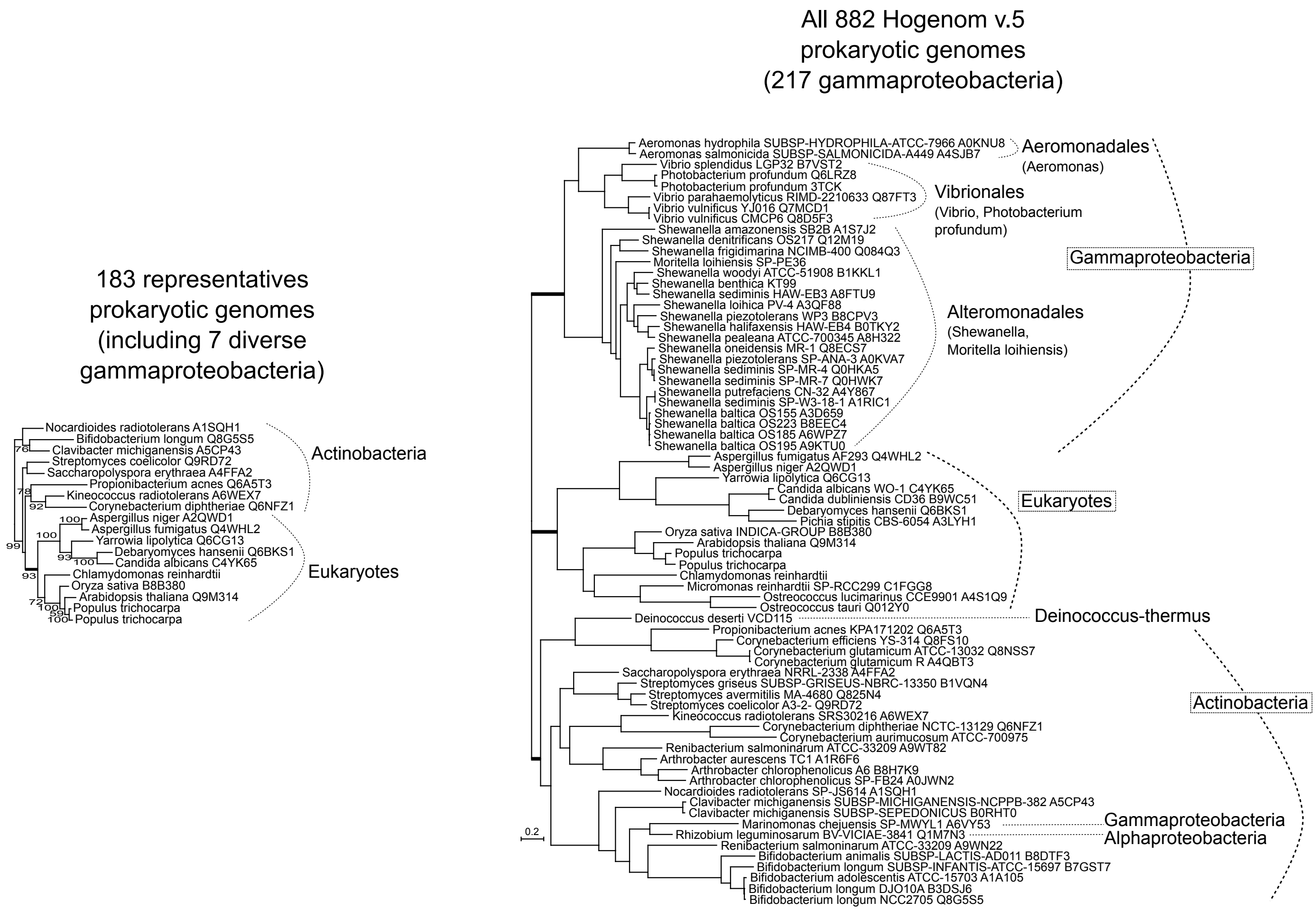

Figure S3

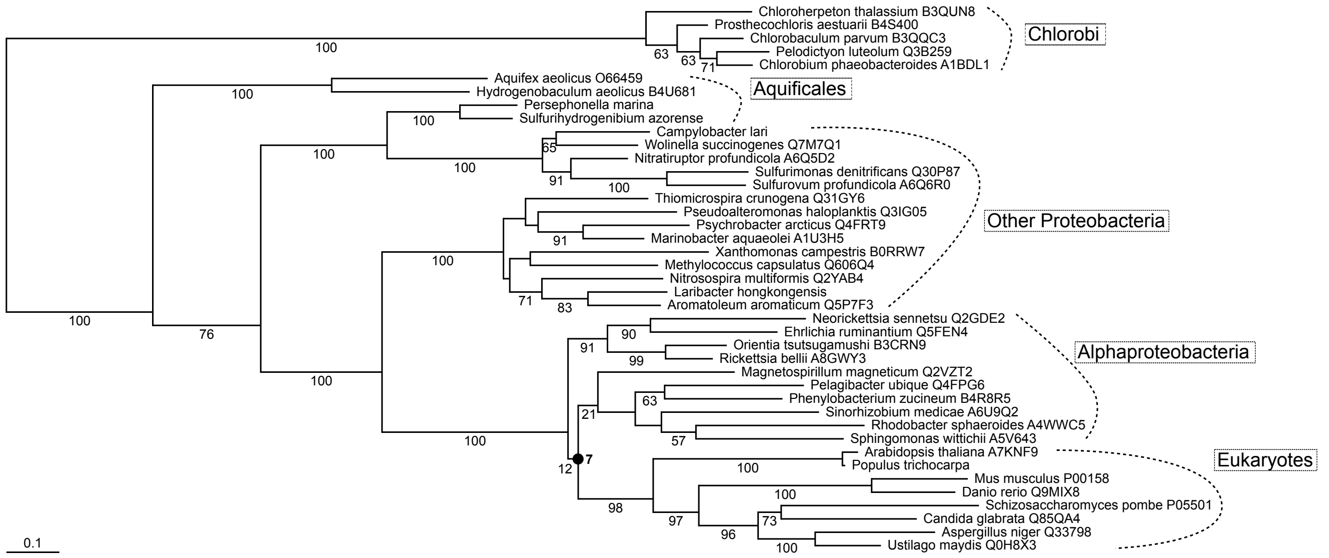

Figure S4. KEGG categories

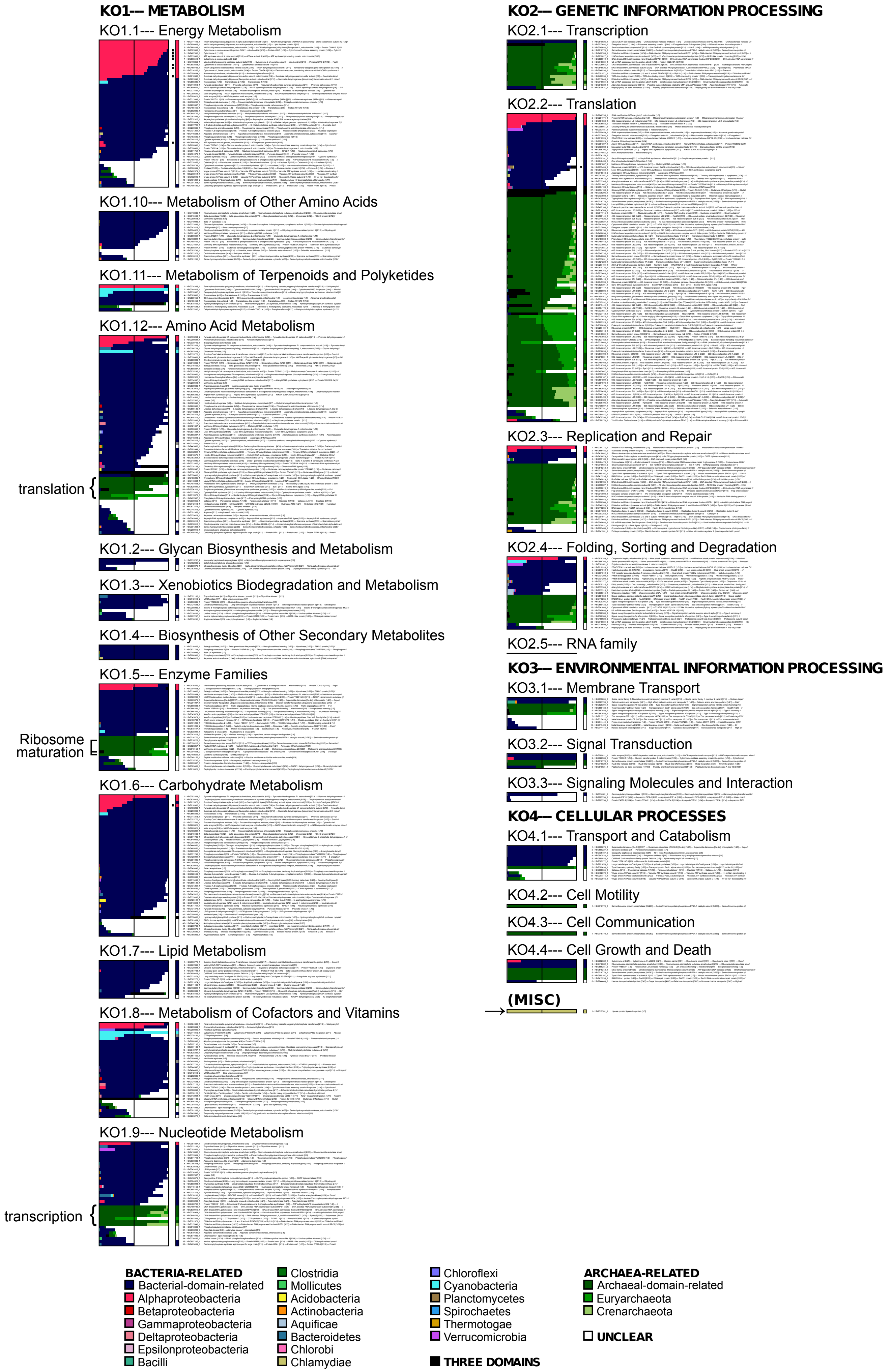

Figure S5

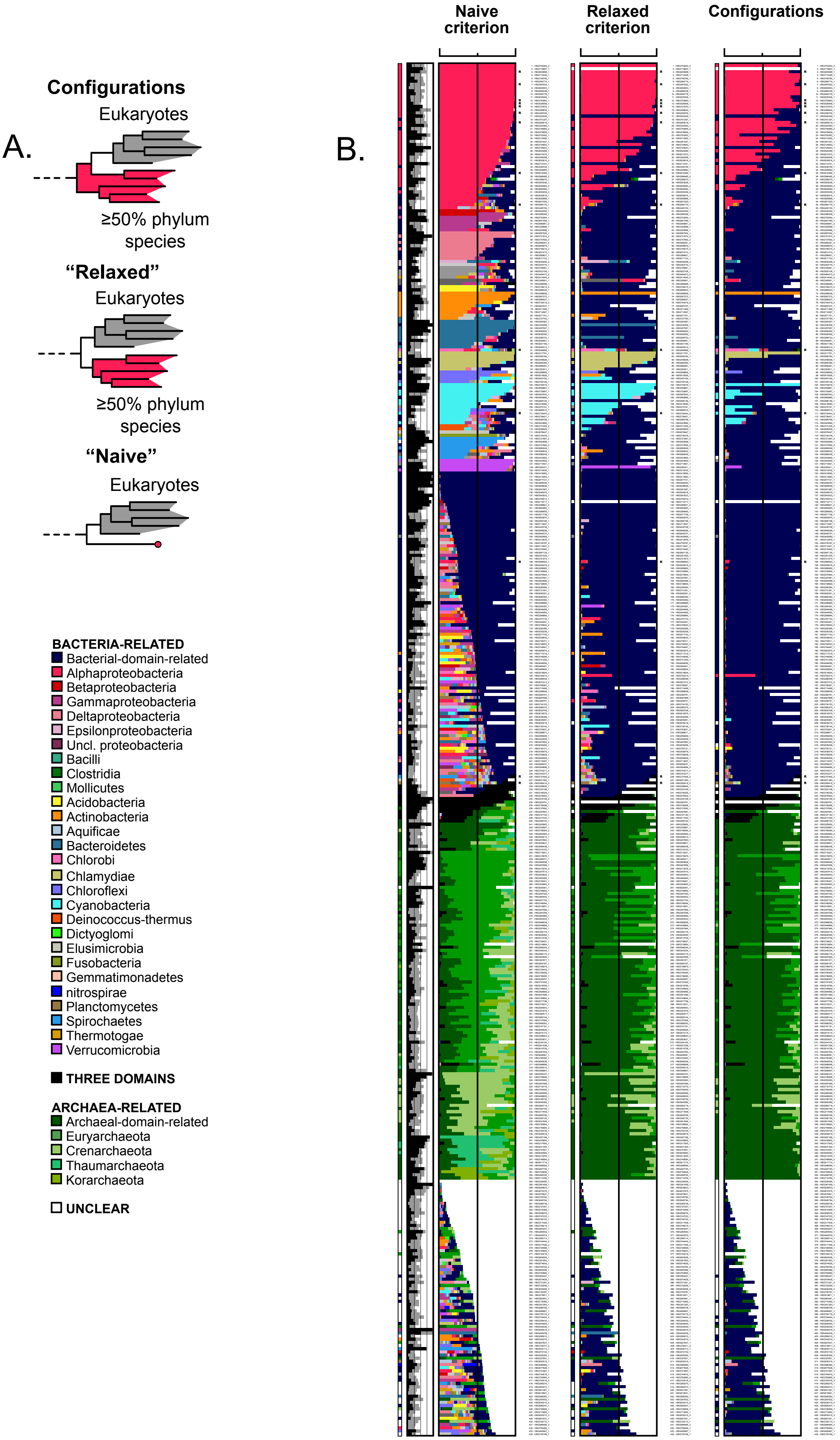
A.
Configurations
Eukaryotes
≥50% phylum species
"Relaxed"
Eukaryotes
≥50% phylum species
"Naive"
Eukaryotes
BACTERIA-RELATED
Bacterial-domain-related
Alphaproteobacteria
Betaproteobacteria
Gammaproteobacteria
Deltaproteobacteria
Epsilonproteobacteria
Uncl. proteobacteria
Bacilli
Clostridia
Mollicutes
Acidobacteria
Actinobacteria
Aquificae
Bacteroidetes
Chlorobi
Chlamydiae
Chloroflexi
Cyanobacteria
Deinococcus-thermus
Dictyoglomi
Elusimicrobia
Fusobacteria
Gemmatimonadetes
nitrospirae
Planctomycetes
Spirochaetes
Thermotogae
Verrucomicrobia
THREE DOMAINS
ARCHAEA-RELATED
Archaeal-domain-related
Euryarchaeota
Crenarchaeota
Thaumarchaeota
Korarchaeota
UNCLEAR
B.
Naive criterion
Relaxed criterion
Configurations

Table S1: List of our 28 near-universal genes and overlap with other studies.

LECA clade	Yeast sequence (Uniprot)	Also used by :		Description
		Guy2011	Williams2012 (GI)	
HBG299153_1	P25443	COG0098	6321315	40S ribosomal protein S2
HBG304663_2	P36132	COG0533	37362674	Probable tRNA threonylcarbamoyladenosine biosynthesis protein KAE1
HBG307515_1	Q01855			40S ribosomal protein S15
HBG317444_1	P38701			40S ribosomal protein S20
HBG385416_1	P06367	COG0100	10383793	40S ribosomal protein S14-A
HBG397258_1	P33322			H/ACA ribonucleoprotein complex subunit 4
HBG439330_1	P04456			60S ribosomal protein L25
HBG457663_1				Lost in yeast ; Q5VV42 (human) : Threonylcarbamoyladenosine tRNA methylthiotransferase
HBG518785_1	P0CX51	COG0103	9755341	40S ribosomal protein S16-A
HBG562580_1	P20424		6325345	Signal recognition particle subunit SRP54
HBG562580_2	P32916		398366427	Signal recognition particle receptor subunit alpha homolog
HBG568696_1	P05740	COG0091		60S ribosomal protein L17-A
HBG584843_1	P53942			Ribonuclease H2 subunit A (RNase H2 subunit A) (EC 3.1.26.4)
HBG586249_2	P42942			Uncharacterized GTP-binding protein YGR210C
HBG592167_1	P0C0W9	COG0094	6325359	60S ribosomal protein L11-A
HBG594170_2	P0CX53	COG0080	6320781	60S ribosomal protein L12-A
HBG594899_2	P0CX41	COG0093	398364725	60S ribosomal protein L23-A
HBG606796_1	P05738	COG0097		60S ribosomal protein L9-A
HBG635083_1	P39730		6319282	Eukaryotic translation initiation factor 5B
HBG727142_1	P02406			60S ribosomal protein L28
HBG735443_1	P0C0W1	COG0096		40S ribosomal protein S22-A
HBG736296_1	P32905	COG0052	6323077	40S ribosomal protein S0-A
HBG737405_1	P0CX55	COG0099		40S ribosomal protein S18-A
HBG737692_1	P32324		6324707	Elongation factor 2
HBG747181_1	P0CX29	COG0048	6325389	40S ribosomal protein S23-A
HBG748739_1	P0CX45		6322171	60S ribosomal protein L2-A
HBG748975_1	P05750	COG0092	398364505	40S ribosomal protein S3
HBG750455_1	P0CX47	COG0186		40S ribosomal protein S11-A
